# Mistletoe treatment in cancer-related fatigue: a case report

**DOI:** 10.1186/1757-1626-2-77

**Published:** 2009-01-22

**Authors:** Kathrin Wode, Thomas Schneider, Ingrid Lundberg, Gunver S Kienle

**Affiliations:** 1 Vidarkliniken, S-15391 Järna, Sweden; 2 Karolinska University Hospital, Södersjukhuset, S-11883 Stockholm, Sweden; 3 Södertälje Hospital, S-15286 Södertälje, Sweden; 4 Institute for Applied Epistemology and Medical Methodology, D-79111 Freiburg, Germany

## Abstract

Cancer-related fatigue (CRF) is a major and very common disabling condition in cancer patients. Treatment options do exist but have limited therapeutic effects. Mistletoe extracts are widely-used complementary cancer treatments whose possible impact on CRF has not been investigated in detail. A 36-year-old Swedish woman with a 10-year history of recurrent breast cancer, suffering from severe CRF, started complementary cancer treatment with mistletoe extracts. Over two and a half years a correspondence was observed between the intensity of mistletoe therapy and the fatigue. Mistletoe extracts seemed to have a beneficial, dose-dependent effect on CRF. Although such effect has also been noted in clinical studies, it has never been the subject of detailed investigation. More research should clarify these observations.

## Introduction

Cancer-related fatigue (CRF) is a highly prevalent condition in cancer patients at all stages. It is a most distressing symptom that substantially impairs quality of life (QoL) and physical and emotional functioning. It disrupts daily activities and has a substantial economic impact. [1-3] It is characterized by persistent tiredness disproportionate to activity, and an increased need to rest; a sustained sense of exhaustion that cannot be completely relieved by rest; and diminished energy, mental capacity, and psychological status [4,5]. Causes are poorly understood and research lags far behind research activities on other cancer-related topics. [1,3] Factors that contribute to its development are: cancer type and cancer treatment, length of time after treatment, other medications, anemia, sleep disorders, nutrition problems, pain, activity level, psychosocial factors and others [6]. CRF can persist for years and is also common in disease-free cancer patients (D-FCP) with a prevalence of up to 41% in breast-cancer patients [7,8]. CRF in D-FCP seems to be more closely connected with psychosocial factors (psychological distress, sleep disorders, level of activity) than to cancer type, cancer treatment and length of time after treatment. Recently a correlation with raised levels of pro-inflammatory cytokines was observed [9]. Hitherto, fatigue has not been regarded as a risk factor for relapse; however, a recent report described low fatigue to be a predictor for longer recurrence-free survival in breast-cancer patients [10]. Therapeutic options are limited. Physical exercise, cognitive therapy and medication can have some beneficial effects [11,12] but seem not to provide sufficient relief, and many patients have a sense of resignation regarding the alleviation of their fatigue [2,3].

Cancer patients often use complementary and alternative medicine (CAM). Among the most frequently applied CAM therapies for cancer are aqueous extracts from European mistletoe *(Viscum album L.)*, originally developed as a cancer remedy in the context of anthroposophic medicine (AM) [13,14]. Biological properties have been extensively analyzed and several pharmacologically active compounds isolated. Mistletoe extracts show highly cytotoxic and growth-inhibiting effects, especially through induction of apoptosis, but they also possess DNA-stabilizing properties in mononuclear cells; they stimulate the immune system (*in vivo *and *in vitro *activation of monocytes/macrophages, granulocytes, natural killer cells, T-cells, induction of a variety of cytokines) and can enhance endorphins. [13,15] Injected in tumor-bearing animals, they display growth-inhibiting and tumor-reducing effects. [13,15] Mistletoe remedies, either alone or in combination with surgery, chemotherapy, radiation or hormone therapy, are applied in all cancer types and all stages of disease, in order to improve QoL and general condition, to reduce side effects of oncological treatment and to improve immuno-suppression, and prolong time to progression and survival. [13] Controlled clinical trials found best evidence of efficacy in relation to improvement of QoL and reduction of side effects of chemotherapy and radiation. Most trials also observed survival benefit, but not beyond critique. Mistletoe is generally well tolerated, with no or only minor side-effects. [14]

Mistletoe-prescribing physicians often observe a marked improvement in fatigue after some months of mistletoe application, and even use fatigue as an indicator for individual adjustment of dosage. Nevertheless, CRF has not so far been addressed as a primary objective in mistletoe studies. [13,14,16] As a sub-dimension of QoL assessment however, positive outcomes on fatigue were noted ([17] and Table 1). For instance, two studies evaluating in-patient cancer treatment in AM hospitals (including mistletoe therapy) observed a significant improvement in fatigue levels. [18,19]

**Table 1:** Clinical trials on mistletoe treatment of cancer that also evaluated influence on fatigue (secondary outcome measure).

Sample size	Study type	Primary study question	Assessment of fatigue/tiredness	Results on fatigue/tiredness*
**Studies on mistletoe application**				

233	RCT (mistletoe vs. Lentinan)	QoL	TCM-score	Improvement and advantage (total TCM score significant)
272	RCT, double-blind (mistletoe vs. placebo)	QoL (GLQ-8, Spitzer uniscale)	1) GLQ-8 2) EORTC QLQ-C30	1) Significant advantage 2) No differences of total score
352	RCT, double-blind (mistletoe vs. placebo)	QoL (FACT-G)	GLQ-8	Significant advantage
399	RCT, open (mistletoe vs. no mistletoe)	Disease-free survival	EORTC QLQ-C30	No advantage
25	Phase II trial (single-arm)	Tumor response	Statement	Improvement
804	Retrolective comparative epidemiological cohort study (mistletoe vs. no mistletoe)	Adverse drug reactions from conventional cancer drugs, disease symptoms, functional capacity, hospitalization	Statement	Improvement and significant advantage
	Retrolective comparative epidemiological cohort study (mistletoe vs. no mistletoe)	Adverse drug reactions from conventional cancer drugs, disease symptoms, functional capacity, hospitalization	Statement	Improvement and significant advantage
1442	Retrolective comparative epidemiological cohort study (mistletoe vs. no mistletoe)	Adverse drug reactions from conventional cancer drugs	Statement	Improvement and significant advantage
1248	Retrolective comparative epidemiological cohort study (mistletoe vs. no mistletoe)	Adverse drug reactions from conventional cancer drugs	Statement	Significant advantage

**Studies on whole-system AM care including mistletoe application**				

120 (44)	Matched pair study (AM versus conventional care)	QoL	EORTC QLQ-C30	Improvement and small advantage
110	Sinlge-arm observational study	QoL	EORTC QLQ-C30	Significant improvement

For references see [17].

* Improvement refers to pre-post difference; advantage refers to difference of pre-post-changes between comparison groups

Abbreviations: RCT: randomised controlled trial; QoL: Quality of life; AM: anthroposophic medicine; TCM: Traditional Chinese Medicine Index; GLQ-8: Global Quality of Life Scale; FACT-G: Functional Assessment of Cancer Therapy-General; EORTC QLQ-C30: European Organization for Research and Treatment Core Quality of Life Questionnaire.

Not considered were indirect measurement of fatigue/tiredness, e.g. "I am forced to spend time in bed". Not included are three studies that also used quality of life evaluation tools including questions on fatigue but that did not present any details on fatigue: two small comparative trials using EORTC QLQ-30, one of which reported an advantage, the other one not. One single-arm trial used SF-36 and reported an improvement.

The following case, drawn from routine clinical practice, describes a disease-free breast-cancer patient suffering from severe CRF, who showed a remarkable response pattern to the application of mistletoe extracts.

## Case presentation

### History and presenting condition

A 36-year-old Swedish woman with a history of recurrent breast cancer and severe CRF presented at Vidarkliniken in Sweden. Vidarkliniken provides integrated AM health care [16,18] in an inpatient and outpatient setting, primarily for patients with cancer, stress-related diseases and chronic pain. The woman had been treated for breast cancer with positive axillary lymph nodes 10 years previously (see Table 2 for details of anamnesis, findings and treatment). Additional to surgical removal she had undergone bilateral oophorectomy (as participant in a clinical trial), and had received adjuvant radiotherapy and hormone therapy which, however, was halted after 4 months because of side effects. Eight years later she started suffering from extreme tiredness, and after one further year a relapse was ascertained: a palpable sternum metastasis, confirmed twice by ultrasound-guided fine-needle biopsy. The metastasis was hormone receptor-negative in contrast to her primary breast cancer. On computer tomography two small changes in the lungs were considered as possible metastasis. The woman received palliative chemotherapy which was halted after 5 cycles because of severe side effects. Subsequently, she underwent radiotherapy of the breastbone. Both treatments had good clinical and radiological result: the sternum metastasis was no longer palpable, follow-up computer tomographies and skeletal scintigrams showed complete remission. Subsequently no further disease activity was observed, either radiologically or clinically.

**Table 2:** Anamnesis, findings and treatment.

Age	State of disease	Treatment
**26**	Diagnosis: Left breast cancer, 1.2 cm Histology: moderately differentiated, ductal invasive, Elston-Ellis grade II, estrogen receptor positive, progesterone receptor pos, S-phase impossible to calculate, 2 of 18 axillary lymphnodes positive, no extranodal growth, benign ovaries TNM-classification: pT1 pN1 pM0	Surgery • Local surgery, not radical • Quadrant resection and axillary surgery, radical • Bilateral oophorectomy Radiotherapy: Local 50 Gy, 2 Gy × 25 Hormone therapy: Tamoxifen 20 mg/day, stopped after 4 months due to side effects (bad mood, lower capacity, sensitivity to noise – the symtoms disappeared when therapy was stopped)
**34**	Onset of severe fatigue	
**35**	Relapse: bone metastasis of breast cancer in sternum, verified by 2 fine-needle biopsies, hormone receptor negative, MIB-1 30%, HER-2 neg. On computer tomography two changes in lungs measuring 6 mm, considered as possible metastasis Complete radiological and clinical remission	Palliative chemotherapy: FEC-60 (Fluorouracil, Epirubicin, Cyclophosphamide) stopped after 5 cycles due to side effects (headache, nausea, pain, anxiety) Radiotherapy: Breast bone 39 Gy, 13 Gy × 3
	Diffuse pain especially in knees	Bisphosphonates, stopped by patient after one month because of lack of improvement
	Reduced fatigue for some months after chemo- and radiotherapy	
**36**	Severe fatigue	10-day rehabilitation in an AM hospital Start of mistletoe treatment
**36–38**	Fatigue of varying intensity Relapse-free	Mistletoe treatment: Individually adapted dosage and repeated breaks (see text and : Figure 1)

Eight months after radiation therapy, the patient attended Vidarkliniken for 10 days of cancer rehabilitation. She was suffering from reduced general condition, with migraine-like headache, dizziness and diffuse pain throughout her body. Fatigue had temporarily improved after radiation therapy, but recurred after some months, having a strong impact on her daily life and forcing her to rest several times each day. Although she felt tired, she was physically active, going for several walks a day with her dog. She slept well and did not feel depressed or anxious. She was forgetful but had no difficulties in concentrating. Physical examination (including sternum) and routine blood samples were normal. Concerning her social situation, she lived as a single mother with her now 14-year-old son, as was already the case at the time of her primary diagnosis 10 years previously. At that time she had experienced some fatigue, but considered this was due to her life situation as a single mother raising a 4-year-old child. She had no education after high school, and had some short-term employment prior to relapse, being sick-listed since then.

### Treatment

During rehabilitation, several AM treatments were applied – medicines, art and physical therapies – and, in particular, a mistletoe treatment was initiated (Iscador^® ^M).

The main goal of the mistletoe treatment was to improve the patient's general condition, QoL and fatigue, and possibly exert a positive influence on the course of the cancer disease. The mistletoe treatment followed mainly general recommendations, i.e. subcutaneous Series 0, Series 1, or Series 2 injections (Series 2 not used in this case): Series 0 means 7 injections at a lower dosage (2 × 0.01 mg/ml, 2 × 0.1 mg/ml, 3 × 1 mg/ml); Series 1 means 7 injections at higher dosage (2 × 0.1 mg/ml, 2 × 1 mg/ml, 3 × 10 mg/ml). Therapy starts with repeated injections of Series 0 (three injections per week, e.g. Monday, Wednesday, Friday) for five weeks, followed by a one-week pause. After this break, depending on the patient's response, either Series 0 is repeated, or the next series commences. Treatment modifications are geared to the patient's reactions (general condition, specific symptoms, skin reaction): one can switch between series; and one can prolong or shorten the intervals within a series, or the pauses after the five-week treatment cycles.

The treatment was well-tolerated by the patient. When discharged from the clinic after 10 days she stated that she felt physically stronger, inwardly calmer and had less pain. After discharge, all other AM therapies and medicines were terminated, but mistletoe therapy was continued at home.

### Outcome

Mistletoe therapy continued for 3 years. As the patient lived a long way from the clinic, further consultations and follow-up were done by telephone. Timing of these contacts was determined by the patient's needs, the intervals varying between weeks and months. At every contact, the physician documented information on mistletoe dosage, general condition and/or fatigue.

During this time, dosage of mistletoe extracts varied substantially. Changes of dosage were usually recommended by the doctor, and recurring temporary treatment interruptions decided by the patient herself. The patient's reasons for interruption were improvement of fatigue to a satisfactory level, and her discomfort with ongoing anti-cancer therapy despite being considered disease-free. Altogether the patient reported a highly variable level of fatigue and overall condition. A close correspondence between mistletoe application, fatigue and general condition emerged. The common pattern was a worsening of overall condition and fatigue during longer treatment breaks or dose reductions, and an improvement after restart or intensification of the mistletoe therapy.

**Figure 1:**
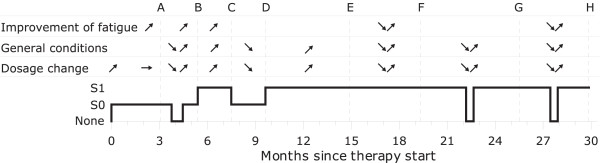
**Changes in mistletoe dosage, fatigue and general condition (pain, nausea, mood, headache).**  ↗ - improvement of symptoms, ↘ - worsening of symptoms, → - no change of symptoms.   S0 – Series 0, S1 – Series 1. Letters (A, B, …) refer to description in text.

For the timing (A, B, ...) of the following description, see : Figure 1:

(A) Follow-up 3 months after rehabilitation period. After a period of varying intensity of fatigue, and repeated perspiration during the initial weeks of mistletoe treatment, the patient continued to inject mistletoe S0 and felt less tired. Mistletoe dosage was now slightly decreased due to local skin reactions.

(B) She reported halting mistletoe injections at her own decision for some weeks, during which she experienced an impairment of her general condition. After restarting the injections, conditions improved: she felt stronger, suffered less headache, no longer had nausea and felt less fatigue. She was satisfied with her overall condition. Mistletoe dosage was now increased from S0 to S1 because of good clinical effect.

(C) She described improved fatigue symptoms as well as better general condition throughout the two months following the last dosage increase. The mistletoe dosage was now decreased from S1 to S0 due to local skin reactions.

(D) She reported that she felt "lifeless" on the lowered dosage S0, and that she had increased general pain. The dosage was now increased to S1.

(E) Next contact after 5.5 months. She reported a better general condition on the higher dosage, but she felt tired on days without mistletoe. The dosage was left unchanged.

(F) She lowered the overall dosage by changing the scheme at her own discretion, taking one-week breaks after every series instead of after every second series. She reported worsening of general condition (pain, nausea, fatigue) during these one-week breaks. General condition and fatigue always improved significantly during subsequent injection periods. In : Figure 1 only one such break is shown to illustrate the correlation. She even noticed a pattern of increasing fatigue on Sundays, and regular improvement of her fatigue on Mondays (injection days Monday, Wednesday and Friday). The mistletoe dosage was now increased by shortening the breaks after every series, from one week to 4 days.

(G) She reported that she felt depressed during longer breaks in mistletoe treatment. After restart, she recovered totally to her former level. Unfortunately, there was no documentation of length and number of these longer, patient-initiated breaks. In : Figure 1 only one such break is shown to illustrate the correlation.

(H) She halted mistletoe injections at her own discretion for two weeks: during this time she felt bad, trembled from physical effort when walking with her dog, and had less energy and more nausea. These symptoms disappeared after restart of the injections, and she again felt as she did before the interruption to treatment.

Altogether, the patient's fatigue could never be resolved completely, her overall capacity remained low, and she remained on sickness benefit. Psychologically, she struggled with the conflicting status of having been in an incurable, palliative state of disease and subsequently being considered disease-free. Continuing to use a cancer therapy (mistletoe extracts) contributed to her confusion, and as, due to her severe fatigue, she did not feel well, she was unsure what to believe.

After 30 months her general condition worsened, with severe back pain, stomach ache, and weight loss. This raised the suspicion of breast-cancer recurrence, but after several radiological and clinical investigations, as well as blood samples, the patient was still considered to be in complete remission. She finally halted mistletoe treatment after a total of 36 months. The reasons for this were: increased perspiration after dose intensification, her ongoing uneasiness with the continued application of a cancer remedy, the dissuasive attitude of her oncologists, and the fact that her family doctor had initiated an intensive treatment program, with physiotherapy and a combined form of occupational and cognitive therapy, in order to help her back to work. This new approach made her feel totally exhausted, and without energy for anything else. Unfortunately, this approach has not so far achieved the desired result.

### Differential diagnoses and concomitant therapies

The diagnosis of CRF was confirmed according to the ICD-10 criteria (Table 3) [4,5]: 11 of the 13 criteria were met, including the main criterion A1. (For confirmation, 6 of the 13 ICD10-criteria including A1 have to be fulfilled [4].)

**Table 3:** Draft ICD-10 criteria for CRF.

**A1**	Significant fatigue, diminished energy, or increased need to rest, disproportionate to any recent change in activity level
**A2**	Complaints of generalised weakness or limb heaviness
**A3**	Diminished concentration or attention
**A4**	Decreased motivation or interest to engage in usual activities
**A5**	Insomnia or hypersomnia
**A6**	Experience of sleep as unrefreshing or nonrestorative
**A7**	Perceived need to struggle to overcome inactivity
**A8**	Marked emotional reactivity (e.g., sadness, frustration, or irritability) to feeling fatigued
**A9**	Difficulty completing daily tasks attributed to feeling fatigued
**A10**	Perceived problems with short-term memory
**A11**	Postexertional malaise lasting several hours
**B**	The symptoms cause clinically significant distress or impairment in social, occupational, or other important areas of functioning
**C**	There is evidence from the history, physical examination or laboratory findings that the symptoms are a consequence of cancer or cancer therapy
**D**	The symptoms are not primarily a consequence of comorbid psychiatric disorders such as major depression, somatization disorder, somatoform disorder, or delirium

CRF is diagnosed when six (or more) of the symptoms above have been present everyday or nearly every day during the same two-week period in the past month, and at least one of the symptoms is significant fatigue (A1) [4,5].

Other diagnoses potentially leading to severe fatigue were largely excluded: no signs of *major depression *were ever revealed by the patient during the whole observation period. Also, in a formal assessment using the Montgomery-Åsberg Depression Rating Scale (MADRS-S), she received 12 out of a possible score of 54, 11 being the "no depression" limit [20]; and in a structured telephone interview following DSM IV criteria for major depression, she did not present depressive symptoms. The patient was in a difficult life situation, and psychological effects probably contributed to her fatigue and general condition. Nevertheless, the patient's overall psychosocial situation was largely stable and therefore did not explain the highly variable fluctuations in her condition. There was also no reason to suspect any further specific *psychological distress *underlying the fatigue [5]. Nor were there signs of *chronic fatigue syndrome *or *burn-out syndrome*. *Hormonally *induced fatigue – early menopause due to oophorectomy – was unlikely due to lack of a temporal link: fatigue started seven years later. *Radiation-induced cardiomyopathy *causing fatigue was also excluded: fatigue had started one year before radiation of the sternum and an echocardiogram two years after radiation showed normal findings. *Hypothyroidism *and *anemia *were also repeatedly excluded

The patient used vitamin B12 preparations from time to time and at her own decision, but did not experience any change in fatigue correlating with its use. In fact, vitamin B12 deficiency was detected after the observation period, but after successful vitamin B12 substitution no change in fatigue was observed. The various treatments applied during rehabilitation at the AM hospital were all discontinued after discharge. The patient did not have a specific exercise program, but went on several walks each day on her own initiative. This, however, did not correlate with the fatigue.

## Discussion

This case is striking because, during a two-and-a-half-year observation period, the patient spontaneously reported highly fluctuating levels of fatigue and general condition that corresponded to the application and dosage of mistletoe treatment: dose reductions (prescribed by the physician) and therapy breaks (decided by the patient) were usually followed by a worsening of fatigue and/or general condition, while increase in dosage or restarting of treatment led to improvement in fatigue and better general condition.

The explanation for these observations can only be hypothetical at the current status of knowledge. Mistletoe therapy has often been reported to improve QoL. [13,14] A number of clinical studies that investigated the influence of mistletoe on QoL also raised the topic of fatigue, and most showed positive results (see Table 1); none of them, however, assessed CRF in D-FCP. The biological factors involved in the modification of fatigue can only be a subject of speculation mainly because the mechanisms responsible for CRF are largely unknown. Endorphins might be involved, since these are enhanced by mistletoe applications; or the cytokine network, which is also influenced by mistletoe extracts (overview see [13,15]). However, other or multiple factors, including more psychological ones, could also be involved.

Irrespective of any causal explanation, the observations in this case are clinically relevant. Fatigue is a major and unsolved problem in and after cancer disease and has a profound effect on QoL. [1,3] It is one of the most common unrelieved symptoms in the cancer context, and affects patients significantly and extensively, more than any other symptom such as anxiety, pain, nausea/vomiting, hair loss, depression, alopecia, etc. [3,21] Patients feel that fatigue is the biggest problem. It affects central aspects of their lives such as the ability to work, to take care of the family, to have relationships with friends, and to enjoy life. [3] Given the magnitude of the problem, it is astonishing that research on CRF is so underdeveloped. Treatment options are limited: they are mainly behavioral, involving exercises and psychosocial interventions, but also some medication. Such treatments do show positive results in reducing fatigue, but with limited clinical significance for the individual patient, and do not seem to be of sufficient help to all patients. Their effect on fatigue in D-FCP largely remains an open question. [6,11,12]. There is an urgent need, therefore, to find further therapy options.

The observations presented here should be interpreted in their context; the case derives from daily practice, and was not specially designed for subsequent reporting. It has major limitations, especially insofar as no well-established questionnaire was used to assess the fluctuations on fatigue and its impact on daily life. Several validated, sophisticated, multidimensional methods would be available to assess fatigue; they are, however, primarily designed as research tools, and are difficult to use in clinical practice [2,6]. For routine use, only simple questions can be a guide for approximately estimating the extent of fatigue and its impact over time (for instance see Table 4). [22] No matter what assessment method is chosen, ultimately one always has to rely on the patient's own report. The medical information provided in this instance should, therefore, sufficiently facilitate a pragmatic judgement of the case history.

**Table 4:** Questions for assessment of fatigue severity and impact over time in routine practice setting with limited time for evaluation [22].

**1**. Are you experiencing any fatigue?	
**2**. If so, how severe has it been, on average, during the past week? (A simple 0–10 rating scale can be used, i.e. 0–3: mild fatigue, 4–6: moderate fatigue, 7–10 severe fatigue)	
**3**. How does fatigue interfere with your ability to function?	

One could object that the doctor's prescription may have influenced the patient's expectations and affected her experience of fatigue and general condition. It was however generally the patient herself who decided to stop the treatment when she felt better, and when she felt stressed by the psychological conflict of using a cancer medicine while considered disease-free. But despite these strong motives, she restarted treatment each time at her own initiative. The effect of a reporting bias on the outcome over more than two years therefore seems to be of minor relevance. Furthermore, the substantial impact and consistent distress of CRF makes it unlikely that it can easily be improved by mere suggestion from the doctor. In addition, reviews of clinical trials found little or no improvements of QoL and of performance status in cancer patients due to suggestive placebo effects. [23]

The presented case offers insight into routine care of a cancer patient with fatigue, and her spontaneously reported impressions. In this respect the case report presents information usually not documented in controlled clinical trials: the patient's suffering, persistence of the fatigue, the misery of its seeming incurability, reduced quality of life, inability to work, and the multidimensional aspects of the situation. Controlled trials on fatigue hardly ever describe these dimensions, although these are essential for patient care. For instance, the RCTs on pharmacological and non-pharmacological interventions in CRF have usually short observation periods; they do not assess long-lasting improvement or even cure, but only, at best, short-term amelioration with limited clinical significance for the individual patient; and they concentrate on fatigue in the context of chemotherapy, radiotherapy or surgery, or in the palliative situation. No conclusions can be drawn from these RCTs about how to treat disabling CRF in disease-free cancer patients (independent of cancer treatments) so as to provide sufficient relief and restoration of normal functioning. [11,12]

Unfortunately, fatigue never disappeared completely in our patient, and fully returned after final discontinuation of mistletoe application. This did however facilitate observation of pattern correspondences. The therapeutic effect of mistletoe seems quite remarkable, considering that no curative and only limited alleviating options appear to exist. From an AM healthcare perspective, the long-distance consultation in this case has to be regarded as suboptimal; personal contact is considered to be vital and could perhaps have enabled more extensive support from the multimodal AM treatment approach – for instance by drawing on art therapy [24] and physical interventions – which might have contributed to more profound improvement.

Future studies on mistletoe treatment of cancer should investigate possible influences on fatigue, especially in D-FCP, using well established fatigue evaluation tools (e.g. [2,6,25]). Analyses should include questions relating to dosage, treatment pause, preparation, and accompanying therapies. When designing relevant studies, one should remember that mistletoe injections sometimes induce short-term tiredness as part of a mild and dose-dependent, flu-like side effect. [13] Besides treatment superiority in comparative trials, adequate and sustained amelioration of the condition of the individual patient should also be carefully investigated. If this theme is to be addressed by the general physician in routine daily practice, heightened awareness and specific documentation (specific questions (Table 4), VAS, diary, etc.) might be useful for recording important observations (e.g. [22]). As CRF is a complex phenomenon, multimodal approaches including non-pharmacological treatments might be more effective than single remedies. This should also be taken into account in further research.

## Conclusion

Mistletoe treatment showed an effect on the severity of CRF symptoms. As CRF is a major complaint in cancer patients, for which few therapeutic options are available, these observations should be investigated further. Possible effects of multimodal treatment approaches should also be clarified.

## Consent

The patient was in full agreement with publication of her case and she read the final version of the paper. Written informed consent was obtained from the patient for publication of this case report. A copy of the written consent is available for review by the Editor-in-Chief of this journal.

## Competing interests

The case presentation was part of a training course in writing reports relating to cognition-based medicine. The course received funding from Christopherus Stiftungsfond, Mahle Stiftung, Software AG Stiftung, Vidarkliniken, Vidarstiftelsen, Wala GmbH, Weleda AG, Zukunftsstiftung Gesundheit. No sponsor had any influence on the design, execution, interpretation and writing of the case report. All authors declare that they have no competing interests.

## Authors' contributions

KW was responsible for the treatment of the patient and the documentation of the case. KW, TS and GK wrote the case report in close co-operation. GK prepared the literature overview on CFR and mistletoe. TS was responsible for the data analysis. IL drafted the literature overview on CFR. All authors read and approved the final manuscript.
